# A unified gene catalog for the laboratory mouse reference genome

**DOI:** 10.1007/s00335-015-9571-1

**Published:** 2015-06-18

**Authors:** Y. Zhu, J. E. Richardson, P. Hale, R. M. Baldarelli, D. J. Reed, J. M. Recla, R. Sinclair, T. B. K. Reddy, C. J. Bult

**Affiliations:** The Jackson Laboratory, RL13, 600 Main Street, Bar Harbor, ME 04609 USA; DOE Joint Genome Institute, Walnut Creek, CA 94598 USA

## Abstract

We report here a semi-automated process by which mouse genome feature predictions and curated annotations (i.e., genes, pseudogenes, functional RNAs, etc.) from Ensembl, NCBI and Vertebrate Genome Annotation database (Vega) are reconciled with the genome features in the Mouse Genome Informatics (MGI) database (http://www.informatics.jax.org) into a comprehensive and non-redundant catalog. Our gene unification method employs an algorithm (fjoin—feature join) for efficient detection of genome coordinate overlaps among features represented in two annotation data sets. Following the analysis with fjoin, genome features are binned into six possible categories (1:1, 1:0, 0:1, 1:n, n:1, n:m) based on coordinate overlaps. These categories are subsequently prioritized for assessment of annotation equivalencies and differences. The version of the unified catalog reported here contains more than 59,000 entries, including 22,599 protein-coding coding genes, 12,455 pseudogenes, and 24,007 other feature types (e.g., microRNAs, lincRNAs, etc.). More than 23,000 of the entries in the MGI gene catalog have equivalent gene models in the annotation files obtained from NCBI, Vega, and Ensembl. 12,719 of the features are unique to NCBI relative to Ensembl/Vega; 11,957 are unique to Ensembl/Vega relative to NCBI, and 3095 are unique to MGI. More than 4000 genome features fall into categories that require manual inspection to resolve structural differences in the gene models from different annotation sources. Using the MGI unified gene catalog, researchers can easily generate a comprehensive report of mouse genome features from a single source and compare the details of gene and transcript structure using MGI’s mouse genome browser.

## Introduction

Generating lists of genes and other genome features in specific chromosomal regions of the reference mouse genome is a common task among biomedical researchers. Although conceptually simple, generating a complete and non-redundant list of genome features can be challenging because there are multiple major independent genome annotation providers that use different methods for predicting genes. Each of these genome annotation processes generates a set of gene models in which some predictions are unique to a particular pipeline. Even when genes are predicted in common, there are often differences in exon structure and inconsistencies in nomenclature. The accession identifiers associated with the predictions also differ among the various providers. As a consequence, a list of genes downloaded from one source does not always match a gene list obtained from a different source. Further, there are a number of annotation projects that specialize in specific types of genome features such as regulatory regions (Yue et al. 2014) and functional RNAs (Chan and Lowe 2009; Kozomara and Griffiths-Jones 2014). These features are often not included in the predictions of the major annotation providers or are represented incompletely.

We describe here the methods we use to combine annotations from multiple sources into a single “unified gene catalog” for the laboratory mouse reference genome. The Mouse Genome Informatics (MGI) unified gene catalog process does not simply append the different sources of mouse genome feature predictions/annotations together; rather, equivalent genome features from different sources are mapped to a single, unique accession identifier and assigned official standardized nomenclature. Genome features from specialty annotation databases such as miRBase for miRNAs (Kozomara and Griffiths-Jones 2014), Rfam for rRNAs (Burge et al. 2013), and gtRNAdb for tRNAs (Chan and Lowe 2009) are also integrated into in the MGI gene catalog. In this report, we focus on the integration of gene models and curated annotations from the three major genome annotation providers: NCBI, Ensembl, and Vertebrate Genome Annotation database (Vega).

**Fig. 1:**
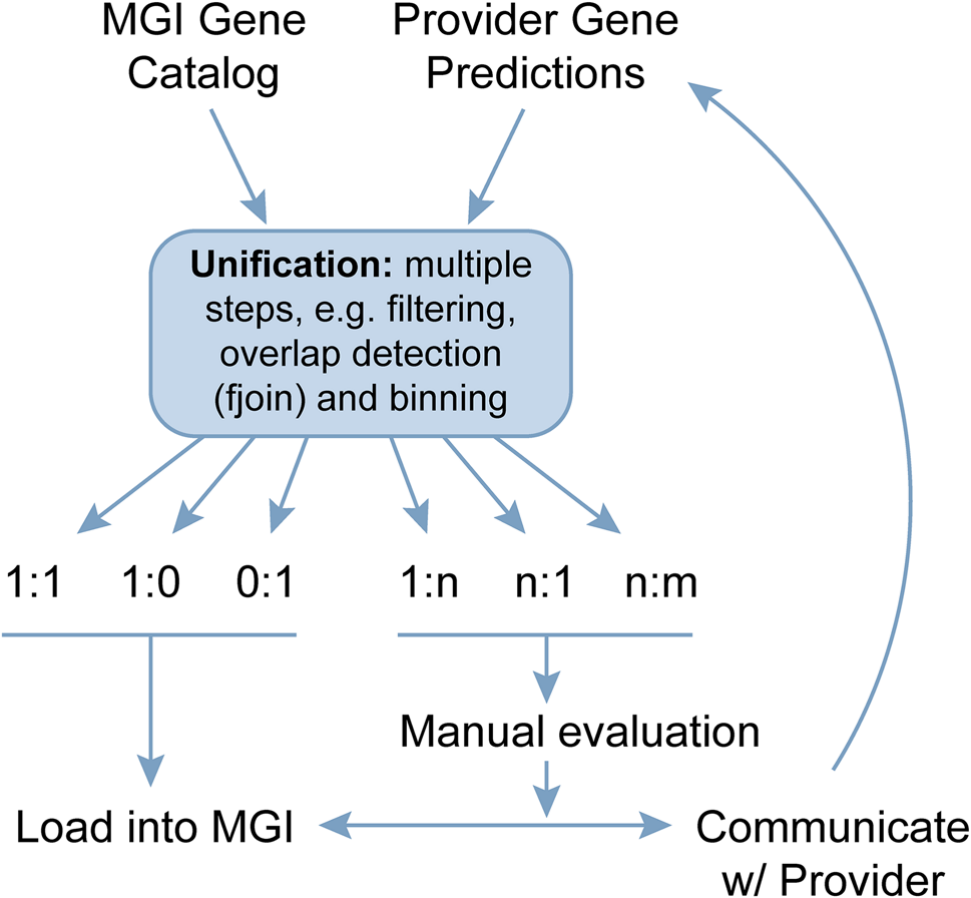
An overview of the gene unification process. Following the comparison of gene predictions and curated annotations using fjoin, the coordinate-based overlap results are binned into six categories. Three of the categories (1:1, 0:1, 1:0) can be loaded into MGI with minimal manual assessment. The other three categories (1:n, n:1, n:m) require manual assessment followed by resolution of annotation discrepancies through communication with the annotation provider(s) or by changes in MGI

The MGI gene catalog is generated using a semi-automated, scalable analysis pipeline called GU (for “gene unification”) that estimates the equivalency of genome features based on genome coordinate overlap. At the heart of this pipeline, there is an algorithm called fjoin (feature join) (Richardson 2006). While a trivial-nested loop can find all pairs of overlapping features in two inputs files, the running time grows geometrically with the file sizes. Fjoin performs the same computation far more efficiently; comparison of two genome annotation files with hundreds of thousands of annotated features takes only minutes to perform. Genome features with overlapping coordinates form bipartite graphs, which are separated and categorized according to the number of participating top-level features. The categories are labeled by cardinality: 1:1, 1:0, 0:1, 1:n, n:1, and n:m (Fig. 1). These groupings make it easier to target gene sets that require manual inspection to resolve annotation discrepancies. The 1:1 category includes instances where a feature in one annotation file overlaps one, and only one, genome feature in the second annotation file, and vice versa. The 1:0 and 0:1 categories include features that are unique to one of the annotation files. The 1:n and n:1 categories include instances where a feature in one annotation file overlaps more than one feature in the other annotation file. The n:m category reflects complex relationships involving multiple features from both annotation files.

The primary inputs for the MGI unified gene catalog are the genome feature predictions and annotations for the laboratory mouse reference genome generated by three major genome annotation providers: Ensembl, NCBI, and Vega. Each of these annotation providers employs different computational and manual methods that result in similar, but not identical, sets of gene models. For mouse, NCBI’s Eukaryotic Genome Annotation Pipeline starts with the alignment of transcripts and proteins, especially sequences that included the curated RefSeq resource (Pruitt et al. 2014). Splign is used for transcript alignment (Kapustin et al. 2008) and ProSplign for protein alignment). The pipeline also incorporates results from an HMM-based gene prediction program (Gnomon) (Pruitt et al. 2014). The Vega database represents clone-by-clone manual curation of finished genomic sequences by the Human and Vertebrate Analysis and Annotation (HAVANA) group at the Sanger Institute (Wilming et al. 2008). Ensembl’s automatic gene annotation system relies on the alignments of mRNAs and protein sequences to the assembly (Flicek et al. 2013). In addition, the Ensembl genome annotation incorporates all of the genes manually annotated by HAVANA group. GU accepts as input annotation files in General Feature Format (GFF or GFF3; http://www.sequenceontology.org/gff3.shtml). Genome features are usually genes, but any entity with genome coordinates can be used as input. GU is highly configurable and the amount of coordinate overlap required to call two features equivalent can be adjusted; the types of genome features to be included or excluded in the analysis can be configured, and a requirement for features to be on the same strand can be turned on or off.

## Materials and methods

### Data sources

The following genome feature prediction and curated annotation sources were used for the analysis: NCBI v104, Ensembl v78, and Vega v58 (Table 1). The annotations in Ensembl included both the computational predictions from the Ensembl genome analysis pipeline and the HAVANA team’s manually curated annotations in the Vega database. The manually curated annotations represent a subset of all genome features predicted in the mouse genome. Regardless, annotations from Vega were analyzed as a separate annotation source as we have previously observed cases where some genome features in Vega are not represented in the combined Ensembl/Vega annotation file.

**Table 1:** Genome feature counts from the annotations of the reference mouse genome by NCBI, Ensembl, and Vega and counts of feature types following the integration of the three annotation sources into the MGI unified gene catalog

	Protein-coding genes	Pseudogenes	Other genome features (non-coding RNAs, etc.)
NCBI v104^a^	22,577	9246	12,533
Ensembl v78^b^	22,032	8031	13,283
Vega v58^c^	15,978	7641	6588
MGI unified gene catalog	22,599	12,455	24,007

The counts exclude genome features on unplaced contigs

^a^ http://www.ncbi.nlm.nih.gov/genome/annotation_euk/Mus_musculus/104/

^b^ http://www.ensembl.org/Mus_musculus/Info/Annotation

^c^ http://vega.sanger.ac.uk/info/website/news.html?id=58&submit=Go

Genome feature predictions from NCBI v104 were based on the reference genome assembly GRCm38.p2 for the laboratory mouse (C57BL/6J). Predictions and annotations from Ensembl v78 and Vega v58 were based on assembly GRCm38.p3. Details regarding the differences in the assembly versions are available from the Genome Reference Consortium (GRC) web site (http://www.ncbi.nlm.nih.gov/projects/genome/assembly/grc/) (Church et al. 2015). Annotation files from Ensembl and Vega were converted from GTF format to GFF3 prior to the GU analysis.

### Gene unification (GU) using the fjoin algorithm

Annotation files in GFF3 format from two genome annotation sources were used as input to the GU process using the fjoin algorithm (Richardson 2006). Pairwise comparison of the annotations from NCBI, Ensembl, and Vega to each other and to the current MGI gene catalog was performed. The fjoin program was configured to consider a genome coordinate overlap of a single-nucleotide position on the same strand as sufficient for establishing the initial assertion of equivalency of two genome features. For protein-coding genes, the fjoin analysis was further constrained to consider coordinate overlaps between exons. We informally evaluated the results obtained from fjoin using different overlap values (e.g., 1, 10, 20, 50, 70, and 100 nt). Only minor differences were noted in the numbers of features in each of the fjoin categories. For example, small RNAs were often included in 0:1 or 1:0 categories when overlaps of more than 20 nt were required leading to false negative equivalency assertions. Changing overlap parameters did not significantly reduce the time needed for manual review of genome features in complex overlap categories (e.g., 1:n, n:1, n:m).

### Processing fjoin categories

Genome features that were deemed to be equivalent to existing MGI genome features (1:1 category) were loaded into MGI without manual review. For genome features novel to MGI (0:1 category), new gene records were created and assigned official nomenclature according to the guidelines of the International Committee on Standardized Nomenclature for Mice.

The genome features that fell into the n:1, 1:n, and n:m categories were reviewed manually. When the manual review identified genome features needing changes to annotations in external resources, the cases were shared with curators at HAVANA and NCBI using a private, on-line Mouse Genome Annotation (MGA) issue tracking system. The MGA allows for coordinated analysis and resolution of annotation discrepancies thereby supporting ongoing refinement and improvement of mouse genome annotations. The MGA resource was implemented using JIRA (https://www.atlassian.com/) and is hosted by NCBI.

### Biotype conflicts

In addition to genome features and their coordinates, the MGI gene catalog also includes the biotype annotations associated with the features in each of the annotation sources. Some of the prediction/annotation sources include biotype annotations for both genome features and the transcripts of those features. We used only genome feature level annotations to identify contradictory biotypes among features our unification process identified as being equivalent; transcript-level biotypes were not considered in the biotype conflict evaluation. As there is no single biotype vocabulary that all annotation groups share in common, we maintain a mapping among the various terminologies in a biotype thesaurus. For example, NCBI annotates all pseudogenes using the biotype term “pseudo.” In contrast, pseudogenes from Vega are classified into multiple subcategories: unitary_pseudogene, processed_pseudogene, translated_processed_pseudogene, transcribed_processed_pseudogene, unprocessed_pseudogene, translated_unprocessed_pseudogene, and transcribed_unprocessed_pseudogene. All of these terms are considered equivalent to the biotype of “pseudogene” in MGI. Similarly, Vega’s IG_pseudogene and TR_pseudogene biotypes and Ensembl’s IG_D_pseudogene, IG_C_pseudogene, IG_V_pseudogene TR_J_pseudogene, and TR_V_pseudogene are all considered equivalent to the term “pseudogenic gene segment” in MGI.

The MGI biotype thesaurus is updated as new biotype terms appear in the annotation files from the three major annotation providers. Within MGI, genome features are given biotype labels based on terms in the Sequence Ontology (Mungall et al. 2011).

### Updating the unified gene catalog

The unified gene catalog is updated whenever a new annotation version is released by NCBI, Ensembl, or Vega. The updates include the addition of new genome features as described above as well as the discontinuation of genes that no longer have evidence to support them. When a new version of the reference mouse genome assembly is released, the coordinates in the unified catalog are converted to the new assembly coordinates using NCBI Remap (http://www.ncbi.nlm.nih.gov/genome/tools/remap).

## Results

### Pairwise comparison of annotations using fjoin

**Fig. 2:**
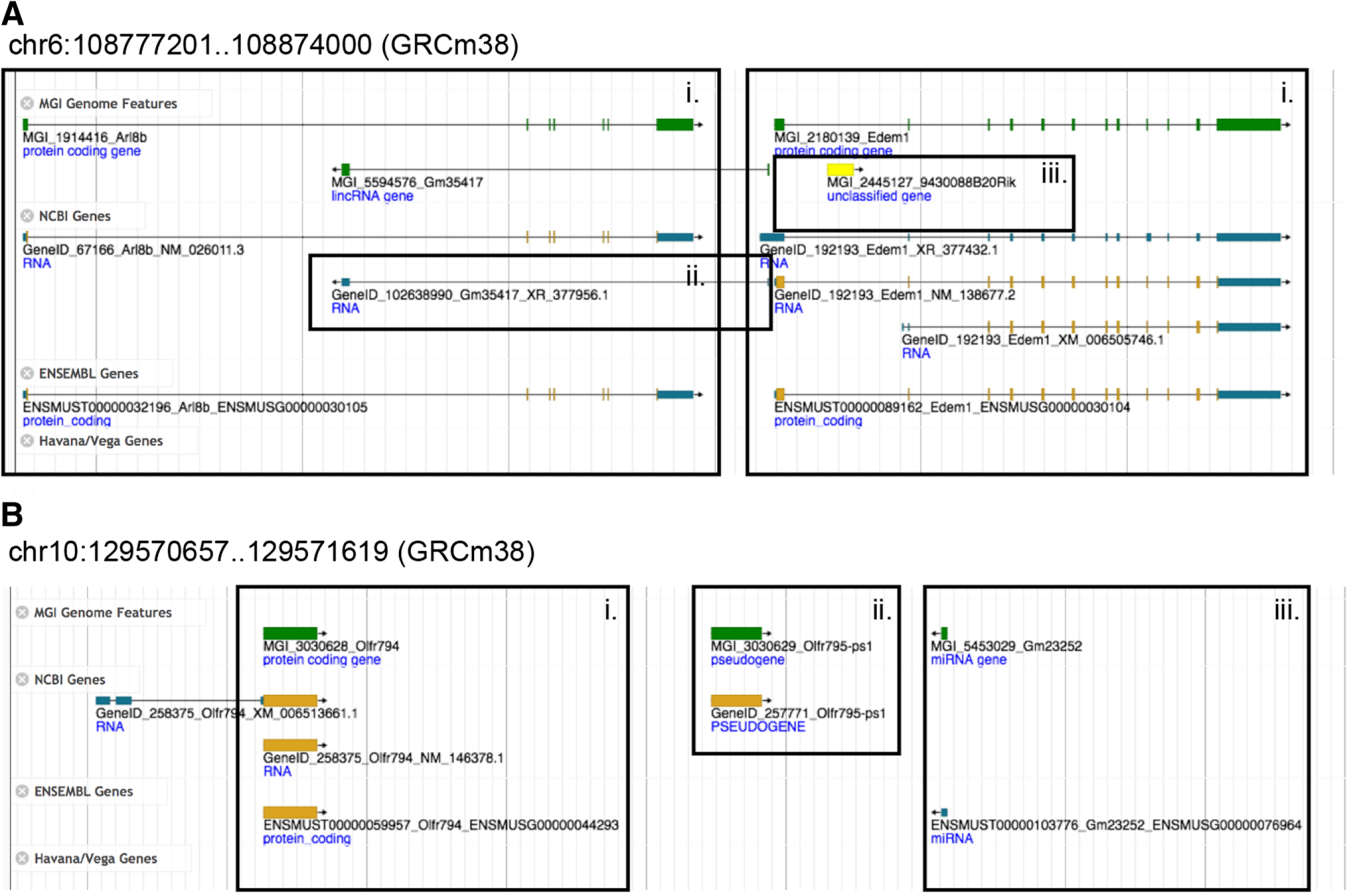
Example of genome features in the 1:1, 1:0, and 0:1 categories generated by fjoin. **a** (*i*) The *Arl8b* and *Edem1* genes have equivalent (1:1) predictions in NCBI and Ensembl, but these genes are not currently represented in the Vega database (1:0). (*ii*) The NCBI non-protein-coding RNA gene (GeneID:102638990) is unique to the predictions from NCBI (0:1). (*iii*) The MGI gene, *9430088B20Rik* (MGI:2445127), is unique to MGI (0:1). **b** (*i*) The *Olfr794* gene (MGI:3030628) has equivalent (1:1) predictions in NCBI and Ensembl, but not in Vega (1:0). (*ii*) The pseudogene, *Olfr795*-*ps1* (MGI:3030629), is only annotated by NCBI. (*iii*) The miRNA gene *Gm23252* (MGI:5453029) is predicted only by Ensembl

The results of the GU process summarized in Table 2 illustrate that the majority of genome features from the various input annotations had equivalent entries in all three sources of genome annotation. However, there were also many features that were unique to a particular source (Table 2). In general, protein-coding gene predictions are largely similar across the three sources whereas the representation of pseudogenes and non-protein-coding genome features is more variable (Fig. 2a, b).

**Table 2:** Summary of gene unification results for NCBI (version 104), Ensembl (version 78), and Vega (version 58)

Genome features equivalent across all sources	23,174
Genome features unique to MGI	3095
Genome features unique to NCBI	12,719
Genome features unique to Ensembl	11,957
Genome features unique to Vega^a^	0
Genome features requiring manual assessment (1:n, n:1, n:m categories)	>4000

^a^The data from Vega represent a subset of all possible genome annotations that are manually curated by the HAVANA team at The Sanger Institute. The annotations from Vega are included in the Ensembl annotation files. As a result, there are usually no annotations unique to Vega

The 3095 genome features that are unique to MGI mostly consist of genes created from full-length cDNAs sequenced as part of the functional annotation of the mouse (FANTOM) initiative (Okazaki et al. 2002). The 12,719 genome features that are unique to NCBI are mostly long non-coding RNA genes and pseudogenes. These distinct features are likely linked to NCBI’s genome analysis for version 104 which incorporated RNA Seq alignments from projects represented in the Sequence Read Archive (Shumway et al. 2010) to assist in gene structure prediction. Similar to NCBI, the 11,957 Ensembl genome annotations not in NCBI are mostly long non-coding RNA genes and pseudogenes. Over 900 of the unique genes in the Ensembl/Vega annotations are located on chromosome Y, which was not well annotated in previous releases. The reconciliation of genome features from Ensembl, Vega, and NCBI with the previous version of the MGI unified gene catalog resulted in 8896 new genome features in MGI. New records in MGI were created for these features, and they were reviewed and assigned official gene nomenclature and biotype annotations.

**Fig. 3:**
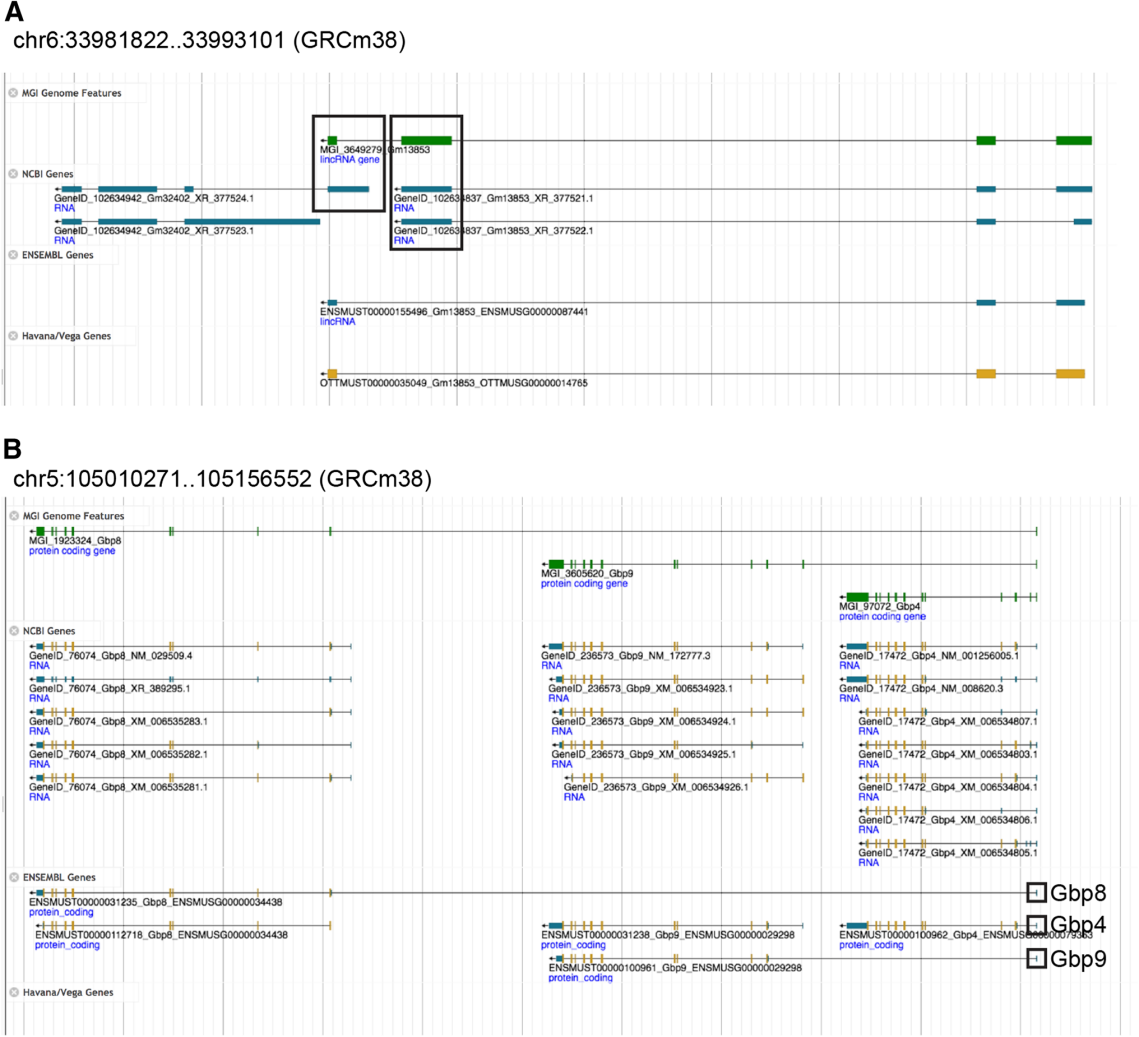
Example of genome features in the 1:n and n:m categories generated by fjoin. **a** The lincRNA gene, *Gm13853* (MGI:3649279), has a 1:n relationship with two NCBI genes (GeneID:102634942 and GeneID:102634837) based on coordinate overlap shown in the boxed regions. **b** The ENSEMBL gene models, *Gbp8* (ENSMUSG00000034438) and *Gbp9* (ENSMUSG00000029298) both have extended first exons that overlap the upstream gene, *Gbp4* (ENSMUSG00000079363) (shown in the boxed regions) resulting in a n:m relationship with the NCBI gene *Gbp4* (GeneID:17472)

**Fig. 4:**
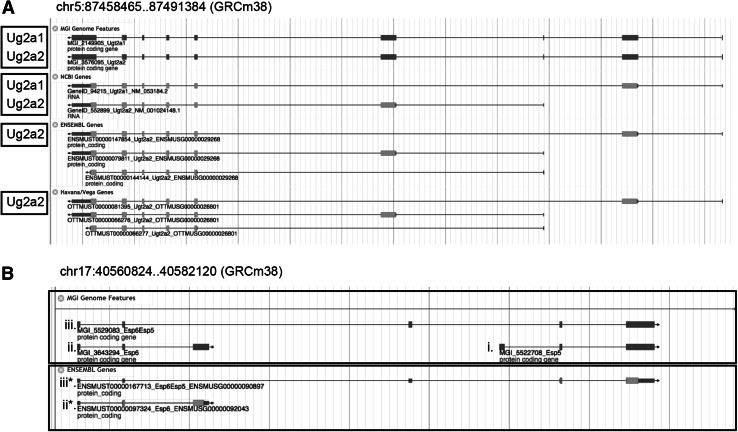
Differences in gene definitions among genome annotation groups lead to ambiguity in determining equivalency of genome features. The cases illustrated in this figure reflect differences in how genes are defined rather than annotation errors and are excluded from further manual review. **a** NCBI and MGI represent *Ugt2a1* (MGI:2149905) and *Ugt2a2* (MGI:3576095) as two different genes while Ensembl and HAVANA represent the data as a single gene with multiple alternative transcripts. **b** NCBI’s mouse genome annotation contains separate entries for (*i*) *Esp5* (MGI:5522708) and (*ii*) *Esp6* (MGI:3643294)as well as the (*iii*) *Esp6Esp5* (MGI:5529083) read through product. Ensembl lacks a specific genome annotation for Esp5, but does represent (ii*) *Esp6* and (iii*) *Esp6Esp5*

**Fig. 5:**
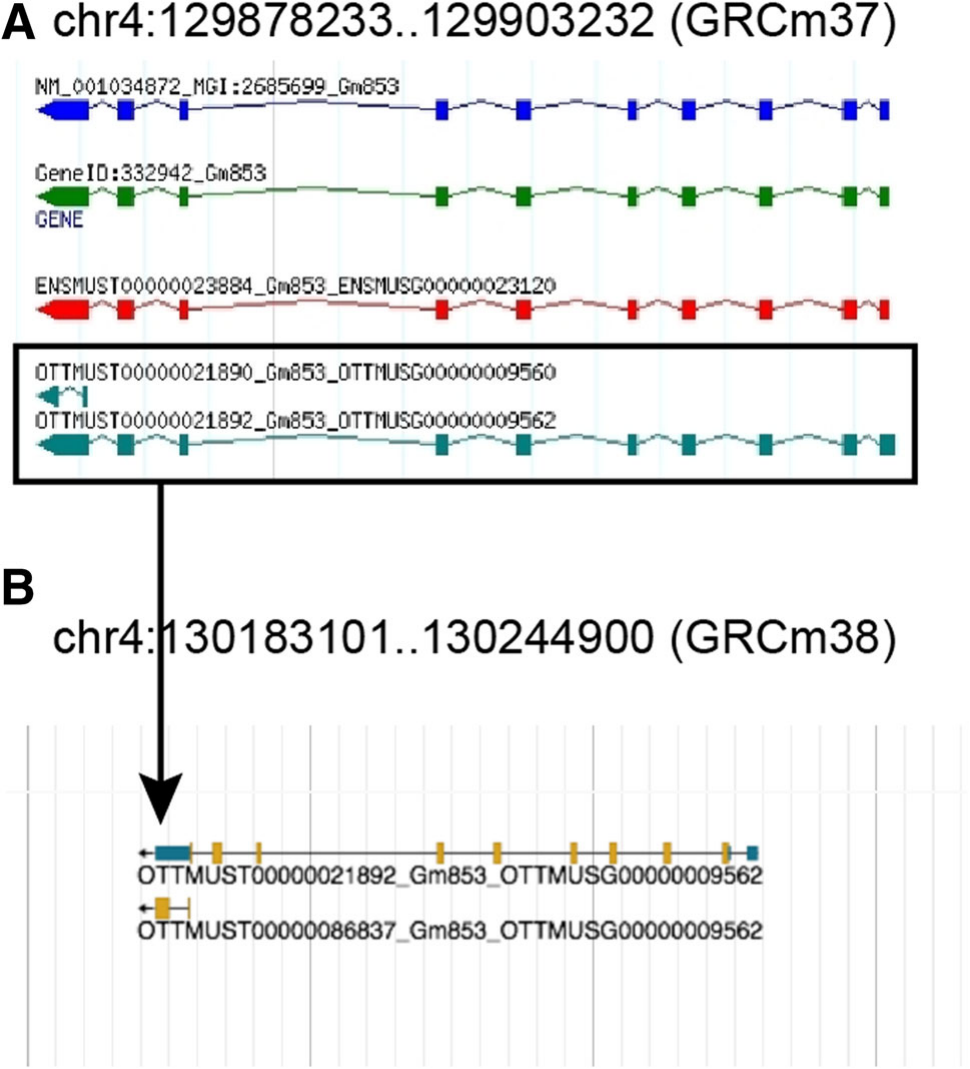
Example of annotation improvements as the results of the collaboration among curators from MGI, NCBI, and Vega. **a** Vega annotation version 35 for the reference mouse genome (GRCm37) included two separate genes (OTTMUSG0000009560 and OTTMUSG0000009562) that overlapped a single gene in the MGI catalog (*Gm853*; MGI:2685699). This case was identified by the review of features in the 1:n category following a previous fjoin analysis. **b** Upon review of all of the evidence, the HAVANA curation team merged gene OTTMUSG0000009560 with OTTMUSG0000009562. The transcript that was previously used as evidence of a different genes is now represented as an alternative processed transcript of OTTMUSG0000009562

Over 4000 genome features from each of the gene prediction and manual annotation providers (NCBI, Ensembl, and Vega) fell in the 1:n, n:1, and n:m categories that need further evaluation (Fig. 3). In many cases, the complex overlaps among features reflect differences in how gene concepts are represented in different databases. For example, *Ugt2a1* (MGI:2149905) and *Ugt2a2* (MGI:3576095) are considered different genes in MGI and NCBI but the Ensembl/Vega groups consider these to be a single gene with multiple transcripts (Fig. 4). MGI database users are alerted to the fact that a genome feature overlaps other genome features by alerts provided in the “Other database links” section of the gene detail page. In yet other cases, the evaluation of genome features in this category identified issues with the gene predictions that required action on the part of the annotation providers. These were shared with the curation groups using the MGA issue tracking system (Fig. 5). The features with complex coordinate overlaps due to differences in gene concepts that appear repeatedly are not reviewed after each new version of the MGI gene catalog is generated. Typically, only about 10 % (several hundred) of the features in the 1:n, n:1, and n:m categories represent new cases that need manual review when the gene catalog is updated.

### Biotype conflicts

**Fig. 6:**
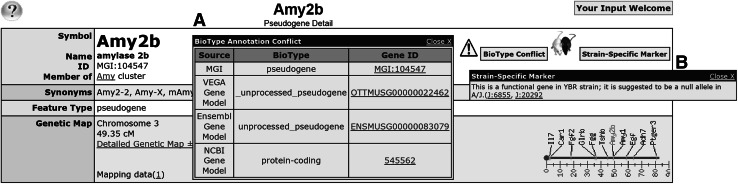
**a** The MGI biotype conflict note is shown for the pseudogene, *Amy2b* (MGI:104547), which is annotated as pseudogene by both Vega and Ensembl but as a protein-coding gene by NCBI. **b** There is also a Strain-Specific Marker notification displayed for this locus because *Amy2b* has been shown to be a functional gene in the YBR strain but a null allele in the A/J mouse strain

There are currently 2086 genes in MGI with biotype conflict note. These cases are highlighted to by the presence of a “biotype conflict” icon displayed on the MGI gene detail page (Fig. 6). One example of a feature with a conflict is the amylase 2b gene (*Amy2b*; MGI:104547). *Amy2b* is a functional gene in YBR strain; it is reported to be a null allele in the A/J inbred mouse strain. (Gumucio et al. 1985; Strahler and Meisler 1982). *Amy2b* is annotated as a pseudogene on the reference genome assembly by both Vega and Ensembl. MGI also annotates *Amy2b* as a pseudogene as there is no direct experimental data for its coding potential in C57BL/6 J. In NCBI, this gene is annotated as a protein-coding gene because according to their annotation guidelines, the biotype “protein coding” applies even if the gene is protein coding in some strains and is a pseudogene in others. A complete list of markers with biotype conflicts is available from the MGI ftp site (ftp://ftp.informatics.jax.org/pub/reports/MGI_BioTypeConflict.rpt).

### Accessing the MGI unified gene catalog

**Fig. 7:**
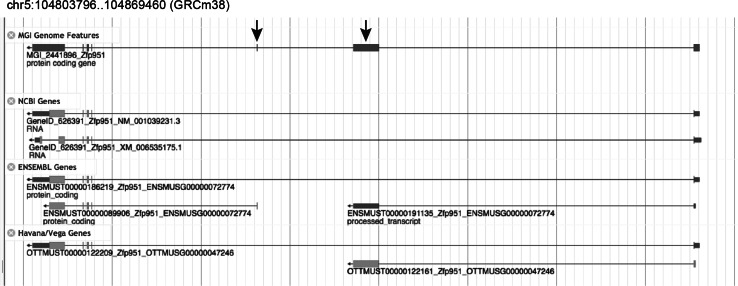
Example of a genome feature in the 1:1 category following fjoin analysis. The *Zfp951* (MGI:2441896) gene has equivalent representations in the annotation output from Ensembl, Vega, and NCBI. However, the structural details of the predictions differ because of how evidence from different transcripts was incorporated into the gene model. The model displayed in the MGI Genome Features track represents an aggregate representation of the gene model components from all three prediction/annotation resources. The arrows highlight features that are present in gene predictions from Ensembl and HAVANA/Vega but not from NCBI

The code and documentation for fjoin is available from MGI’s ftp site (ftp://ftp.informatics.jax.org/pub/fjoin/). The MGI unified gene catalog is searchable via the MGI database (http://www.informatics.jax.org). The MGI gene catalog is also displayed in context of the annotations from Ensembl, Vega, and NCBI using MGI’s JBrowse-based genome browser (http://jbrowse.informatics.jax.org). Within JBrowse users can view the details of similarities and differences of gene structure details across different annotation sources. Each genome feature in the MGI catalog is an aggregate representation generated by combining the annotations from multiple predictions into a single model (see Fig. 7). The annotations are available for download as a tab-delimited file from the MGI ftp site (ftp://ftp.informatics.jax.org/pub/reports/index.html#seq) and as a GFF3 formatted file (ftp://ftp.informatics.jax.org/pub/mgigff/). Also available at the ftp site is the aggregate genome feature file (MGI.exome.gff3.gz) that is used as the basis for the MGI genome feature track in MGI’s genome browser.

Researchers who wish to report an issue or suggested correction for specific mouse genome annotations can submit a report using the public MGA web site (http://www.ncbi.nlm.nih.gov/genome/guide/mouse/MGAReport.shtml). Submissions from the web site results in a “ticket” in the tracking system shared by the mouse annotation curation groups at The Jackson Laboratory, The Sanger Institute, and NCBI.

## Discussion

In this report, we describe the method by which we combine annotation outputs from multiple, independent genome analysis pipelines into a unified gene catalog for the mouse reference genome. As annotated genome assemblies for other mouse strains are generated, we will use the methods described in this report to generate additional strain-specific gene catalogs. The algorithm that drives the GU process, fjoin, is organism-agnostic and could be used to support similar annotation integration efforts for any organism for which there are multiple sources of genome feature predictions.

The MGI unified gene catalog effort has similarities to the Consensus CDS (CCDS) project (to which MGI is a contributing partner) at NCBI (Farrell et al. 2014). Similar to the CCDS initiative, the primary inputs for MGI’s gene catalog are genome annotations from Ensembl, NCBI, and Vega. The CCDS focuses on those annotations/predictions with consistently annotated full-length coding regions (i.e., those with an ATG and valid stop-codon) that can be translated using consensus splice sites without frameshifts. Where the goal of the CCDS is to identify the highest confidence protein-coding gene annotations only, MGI’s gene catalog includes all genome annotations, regardless of biotype. Gene models categorized as equivalent by our unification process are likely to be representations of the same gene or transcription unit. Equivalent gene models, however, are not necessarily identical in gene structure and our pipeline does not evaluate which gene model is likely to be the “best” representation.

The unified gene catalog serves as the foundation for the annotation of biological attributes (i.e., phenotype, function, expression, and pathway membership) of mouse genes by expert curators and bio-data analysts at the MGI (http://www.informatics.jax.org) database (Eppig et al. 2015). The MGI gene catalog also serves as the basis for mouse genome features represented at NCBI’s Gene resource (Brown et al. 2015; Sayers et al. 2012) and was a primary source of genes for the first phase of The International Knockout Mouse Project (KOMP) (Bradley et al. 2012; International Mouse Knockout et al. 2007).

The output from MGI’s unified gene catalog process systematically identifies gene models that are potentially problematic in their structural details as well as those that appear to be equivalent across different sources but have contradictory biotype annotations. The ongoing assessment of genome annotation issues at MGI in cooperation with the international mouse genome annotation community ensures that the biomedical community gains maximum benefit from the reference mouse genome.
